# Prevalence of chronic comorbidities in people with multiple sclerosis: descriptive study based on administrative data in Tuscany (Central Italy)

**DOI:** 10.1007/s10072-022-06345-9

**Published:** 2022-08-18

**Authors:** Daiana Bezzini, Elisa Gualdani, Matilde Razzanelli, Mario Alberto Battaglia, Rosa Cortese, Paolo Francesconi, Monica Ulivelli

**Affiliations:** 1 Department of Life Sciences, University of Siena, Siena, Italy; 2 Agenzia Regionale Di Sanità Della Toscana, Florence, Italy; 3 Research Department, Italian Multiple Sclerosis Foundation, Genoa, Italy; 4 Department of Medicine, Surgery and Neuroscience, Policlinico Le Scotte, University of Siena, Siena, Italy

**Keywords:** Multiple sclerosis, Chronic comorbidities, Prevalence, Administrative data, Sex-related differences, Age-related differences

## Abstract

**Objective:**

Chronic comorbidities are common in people with multiple sclerosis (PwMS), thus worsening their prognosis and quality of life, and increasing disease burden. The aim of the present study was to evaluate the prevalence of common comorbidities in PwMS in Tuscany (Central Italy) and to compare it with the general population.

**Methods:**

The prevalence of comorbidities, including diabetes, chronic obstructive pulmonary disease (COPD), hypertension, stroke, heart failure (HF), cardiac infarction and ischemic heart disease (IHD), was assessed in PwMS and in general population resident in Tuscany, aged > 20 years, using administrative data.

**Results:**

In total, we identified 8,274 PwMS. Among them, 34% had at least one comorbidity, with hypertension being the most common (28.5%). Comparing PwMS with the general population, PwMS had a higher frequency of hypertension and stroke when considering the whole group, and of diabetes, COPD, and IHD when considering sex and age subgroups. This increased risk was especially evident in the young and intermediate age groups, where multiple sclerosis may play an important role as risk factor for some comorbidities.

In PwMS, as well as in the general population, prevalence of chronic diseases was higher in males and increased with age.

**Conclusions:**

Comorbidities frequently coexist with multiple sclerosis and they may have an impact on this complex disease, from the health, clinical, and socioeconomic points of view. Therefore, a routine screening of chronic comorbidities should be a crucial step in clinical practice, as well as the promotion of healthy lifestyles to prevent the onset and to reduce their burden.

## Introduction

The identification of comorbidities (i.e., the presence of one or more chronic illness other than the disease of interest) has a key role in the management of people with multiple sclerosis (PwMS), as they can worsen the prognosis and the quality of life (QoL), influence disability status, healthcare utilization and hospitalization, treatment decisions and response, and increase the risk of death [1]. Comorbidities are common in PwMS due to the disease itself or related to the side effects of drugs [1], or to behavioral risk factors, such as smoking, physical inactivity, obesity, and unhealthy food intake [2]. Further, other chronic diseases may be more easily diagnosed in PwMS due to higher number of follow-up and a widespread use of the health service than the general population [3].

The prevalence of these conditions worldwide varies widely depending on the comorbidity considered, the specific patient population evaluated, and other factors such as demographic and genetic characteristics, socioeconomic condition, and geographic origin [3]. In 2015, an international review identified the most prevalent conditions, which were depression (23.7%), anxiety (21.9%), hypertension (18.6%), hyperlipidemia (10.9%), and chronic lung disease (10.0%) [1]. The authors concluded that there are only a few studies assessing the prevalence of concomitant diseases despite their importance for patients’ management and for the public health planning. Moreover, the majority of these studies are conducted in North America and Western Europe [1], while to date, there are only a few studies in Italy on the prevalence of comorbidities in PwMS. Tuscany is a high risk zone for multiple sclerosis (MS) [4], with a prevalence rate of 208.7 cases per 100,000 in 2017 [5] and an incidence rate of 6.6 cases in 2015 [6]. The evaluation of the concomitant presence of comorbidities and MS in this region of Central Italy has not been performed yet.

Among the currently available sources to capture comorbidities, such as clinical records, patients’ interviews, and administrative databases, the latest are the most cost- and time-saving, as they cover the whole population for a long period, and they are routinely collected, especially in countries with a publicly funded national healthcare system (NHS), such as Italy. However, being collected for the health system management and for the reimbursement by the NHS, and not for research purposes, these data need to be validated before their utilization [7].

In this study, we aimed to (i) assess the prevalence of comorbidities in PwMS in Tuscany, as at 1st of January 2019, using case-finding algorithms based on administrative data, and (ii) compare it with the general population. In particular, although many comorbidities are reported in PwMS, we selected some common chronic diseases, such as diabetes, hypertension, stroke, heart failure (HF), cardiac infarction, ischemic heart disease (IHD) and chronic obstructive pulmonary disease (COPD), because they are routinely monitored, through case-finding algorithms based on administrative data, by the Regional Health Agency of Tuscany to evaluate the public health.

## Methods

### Data sources

Healthcare in Italy is universal, public, and regionally administrated. In observance of the privacy law, data were obtained from the Tuscan Health Administrative database that collects data for the health system management and for the reimbursement by the NHS, and covers the entire regional population (population aged more than 20 years at 1/1/2019: 2,917,181 with 1,379,469 males and 1,537,712 females). Further, the Regional Health Agency monitors administrative database for epidemiological surveillance of several chronic diseases and for control of quality of healthcare [8].

Main sources of administrative database are:

- Inhabitant registry with demographic information of all Tuscan citizens,
- Hospital discharge records (HOSP) reimbursed by the healthcare system, recorded with one main and up to 5 secondary diagnoses coded using the International Classification of Diseases, Ninth Revision (ICD9), and available from 1999,
- Exemption registry (EXE) collecting disease-specific exemptions from co-payment to the healthcare system, recorded with a ICD9 disease code,
- Drug dispensing registry (DRUG) that collects data of drugs with a medical prescription, dispensed directly by local health authorities to community-dwelling patients and/or by private or public pharmacies (available from 2004 and 2003, respectively). Drugs are coded using Anatomic Therapeutic Chemical classification system (ATC),
- Home and residential long-term care (LONG) coded with ICD9 disease code and available from 2010.

### Study populations

To capture PwMS, it was applied a previous validated case-finding algorithm that used population-based, anonymized, administrative health data in which HOSP, EXE, DRUG, and LONG records were linked to inhabitant registry using the unique personal identification numbers that identify all Italian citizens [9].

The cohort of PwMS was composed by all inhabitants alive at the prevalence day who met at least one of the following criteria: (A) at least one HOSP record with MS diagnosis (ICD9CM 340), (B) one EXE record at 1/1/2019 for MS, (C) at least two DRUG records with different dates for at least one of the eight drugs that are specific for MS (see Table 1), and (D) MS diagnosis in LONG database. Data were analyzed from the beginning of the recording of administrative data to the index date (1/1/2019), except for disease-specific payment exemptions regarding only the year before the prevalence date.

**Table 1:** List of drugs specific for MS used for our algorithm and their ATC code

Pharmacophore	ATC code
Glatiramer acetate	L03AX13
Interferon beta 1A	L03AB07
Interferon beta 1B	L03AB08
Fingolimod	L04AA27
Natalizumab	L04AA23
Dimethyl fumarate	N07XX09
Peginterferon beta-1a	L03AB13
Teriflunomide	L04AA31

After identifying the MS cohort, patients aged under 20 years were excluded due to the minimum risk to present comorbidities in younger subjects and to compare these results with other published studies.

Similar algorithms, created and routinely used by the Tuscany Regional Health Agency to monitor other chronic diseases, were used to find in the general Tuscan population the cohorts of people affected (see Table 2). Then, patients’ groups were linked to identify our study cohort.

**Table 2:** Algorithm used for comorbidities

Disease	Algorithm (inclusion criteria)	Validation
Diabetes	(A) at least one HOSP record with diagnosis of diabetes (ICD9CM 250*) AND/OR (B) one EXE record at 1/1/2019 for diabetes, AND/OR (C) at least two DRUG record with different dates for drugs specific for diabetes (ATC A10), AND/OR (D) diabetes diagnosis LONG database	Positive predictive value (PPV) = 100% [36]
Stroke	(A) at least one HOSP record with primary diagnosis code 430, 431, 432, 432, 436 AND/OR (B) ICD9CM 430, 431, 432, 434, 436 diagnosis in LONG database	No validation
Heart failure (HF)	(A) at least HOSP record with diagnosis of HF (428*, 39,891, 40,201, 40,211, 40,291, 40,401, 40,403, 40,411, 40,413, 40,491, 40,493) AND/OR (B) one EXE record at 1/1/2019 for ICD9CM 428, AND/OR (C) ICD9CM 428 diagnosis in LONG database	PPV = 55% [36]
Hypertension	(A) at least one HOSP record with one of these diagnosis codes 401*, 402*, 403*, 404*, 405*, 36, 211 AND/OR (B) one EXE record at 1/1/2019 for 401, 402, 403, 404, 405, AND/OR (C) at least two DRUG records with different dates for drugs specific for hypertension (ATC C09*, C02*, C07*, C08C), AND/OR (D) hypertension diagnosis (ICD9CM 401*, 402*, 403*, 404*, 405*) in LONG database	PPV = 100% [36]
COPD	(A) at least one HOSP record with diagnosis of ICD9CM 490*, 491*, 492*, 494*, 496*, AND/OR (B) DRUG records with different dates for drugs with a code ATC R03*, in particular: •Patients with 45 years of age or more, with more than 120 days between the first and the last prescription, at least 5 packs •Patients with 45 years of age or more, only one therapeutic class with an interval between the first and the last prescription ranging from 30 to 120 days, with a number of prescriptions ranging from 3 to 10 •Patients with 45 years of age or more, only one therapeutic class with an interval between the first and the last prescription ranging from 120 to 210 days, with a number of prescriptions ranging from 3 to 4 AND/OR (D) a diagnosis with one of the code 490*, 491*, 492*, 494*, 496* in LONG database	No validation
Cardiac infarction	(A) at least one HOSP record with diagnosis of ICD9CM 410*	No validation
Ischemic heart disease (IHD)	(A) at least one HOSP record with diagnosis of ICD9CM 410*-414**, AND/OR (B) one EXE record at 1/1/2019 for ischemic heart disease (ICD9CM 414), AND/OR (C) at least two DRUG record with different dates for nitrates (ATC C01DA*), AND/OR (D) a diagnosis with one of the code 410–414 in LONG database	No validation

### Statistical analysis

Age and sex-standardized prevalence of comorbidities were calculated among PwMS and general population using the 2011 Tuscan population (last available population census) as standard. In order to establish the statistical significance of the differences in results obtained in PwMS versus general population, 95% confidence intervals (CI) were computed assuming a Poisson distribution.

A Venn diagram was obtained to show the interconnection of MS and other comorbidities in Tuscan population. We created four elements: MS, COPD, cardiovascular risk (including diabetes and hypertension), and cerebro-cardiovascular diseases (including stroke, heart failure, cardiac infarction, and ischemic heart disease).

## Results

**Fig. 1:**
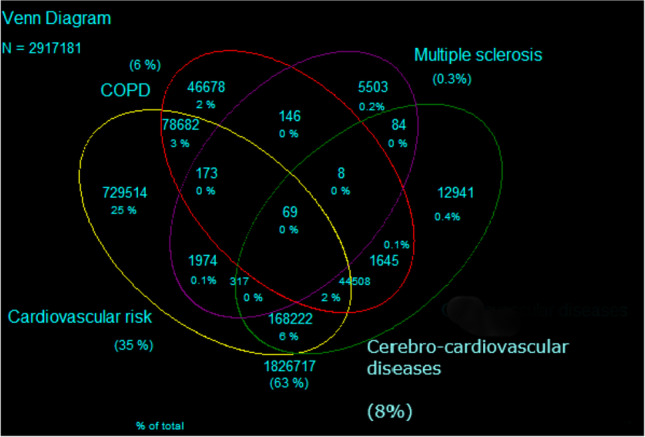
Venn diagram showing chronic diseases among Tuscan inhabitants, aged more than 20 years, as at the prevalence date

As at 1st January 2019, 63% of the Tuscan population aged more than 20 did not have any of the analyzed chronic diseases, whereas 35% had diabetes and/or hypertension (cardiovascular risk group), 8% cerebro-cardiovascular diseases (including stroke, heart failure, cardiac infarction, and/or ischemic heart disease), and 6% had COPD. Around 10% of inhabitants had more than one comorbidity (Fig. 1).

Regarding MS, we identified 8,274 cases with a crude prevalence of 283.6 per 100,000 ab among population aged more than 20.

Among MS prevalent cases, we found 5,503 (66.5%) patients with no comorbidities, 2,354 cases (28.5%) with hypertension, 535 (6.5%) with diabetes, 396 with COPD (4.8%), 257 with IHD (3.1%), 201 (2.4%) with stroke, 122 (1.5%) with cardiac infarction, and 108 with HF (1.3%). Five hundred and sixty-seven PwMS (6.9%) had more than one comorbidity.

When analyzing age and sex-standardized prevalence rates of comorbidities among PwMS, the most common was hypertension with a prevalence of 246.5 per 1,000 followed by diabetes (61.8), COPD (58.1), and IHD (26.5), whereas stroke, cardiac infarction, and heart failure accounted for less than 20 cases per 1,000 MS patients (Table 3). When comparing these rates with the general population and considering all age groups together, we observed statistically higher prevalence rates in PwMS for stroke in both sexes, for diabetes and COPD only in females, and for hypertension only in males, whereas cardiac infarction was more common in general population for both sexes, and heart failure only in female (Table 3).

**Table 3:** Standardized prevalence rates (per 1000 persons) of chronic diseases as at 1/1/2019, in PwMS and general population, according to sex and age group, and relative 95% confidence interval (CI 95%)

	Hypertension		Stroke		IHD		Diabetes		Heart failure		COPD		Cardiac infarction	
	MS (CI 95%)	GEN (CI 95%)	MS (CI 95%)	GEN (CI 95%)	MS (CI 95%)	GEN (CI 95%)	MS (CI 95%)	GEN (CI 95%)	MS (CI 95%)	GEN (CI 95%)	MS (CI 95%)	GEN (CI 95%)	MS (CI 95%)	GEN (CI 95%)
F:20–44	**65.7 (50.5–80.7)**	**25.7 (21.3–28.1)**	**5.7 (3.2–8.2)**	**1.2 (0.8–1.7)**	3.9 (1.5–6.3)	1.0 (0.4–1.5)	**24.5 (20.5–27.7)**	**10.2 (7.3–13.7)**	0.4 (0.1–0.8)	0.4 (0.2–0.7)	3.1 (1.0–4.9)	2.4 (1.6–3.3)	0.5 (0.3–0.8)	0.2 (0.1–0.3)
F:45–59	**222.3 (207.6–237.4)**	**135.8 (132.4–139.0)**	**8.9 (6.4–11.4)**	**4.3 (2.5–6.2)**	**11.3 (8.6–14.1)**	**6.2 (4.3–8.4)**	32.7 (28.3–36.4)	31.4 (25.2–37.8)	3.5 (1.6–5.7)	2.3 (0.4–4.2)	**46.5 (44.7–48.9)**	**32.5 (30.5–34.6)**	4.2 (2.3–6.4)	2.3 (1.2–3.5)
F:60 +	493.2 (478.4–508.6)	523.6 (503.3–545.1)	**39.3 (36.8–41.8)**	**27.8 (24.7–31.6)**	**51.2 (48.7–53.8)**	**78.9 (65.4–91.4)**	124.4 (120.6–128.2)	128.5 (120.1–137.2)	**28.7 (26.4–30.5)**	**36.4 (34.3–37.8)**	**94.6 (92.3–97.2)**	**102.7 (100.6–104.8)**	23.4 (21.4–25.6)	23.6 (20.3–26.7)
Tot F	235.8 (221.8–248.4)	218.7 (208.1–226.6)	**17.7 (12.4–21.9)**	**9.7 (7.3–11.6)**	19.0 (13.7–22.5)	25.3 (21.6–29.2)	**58.6 (52.6–65.7)**	**50.1 (49.8–51.4)**	**8.6 (6.2–10.5)**	**17.8 (14.5–20.3)**	**57.6 (52.3–62.9)**	**50.5 (48.1–51.9)**	**8.3 (7.5–9.5)**	**11.6 (9.8–13.6)**
M:20–44	**58.2 (43.3–73.4)**	**28.4 (24.3–32.2)**	**4.3 (1.8–6.8)**	**1.5 (1.2–1.7)**	**5.7 (3.4–8.2)**	**2.0 (1.5–2.6)**	**23.6 (19.3–27.5)**	**8.7 (6.7–10.3)**	0.9 (0.5–1.4)	1.0 (0.8–1.3)	7.1 (5.2–9.4)	3.8 (1.8–5.7)	2.6 (0.7–4.5)	1.1 (0.9–1.3)
M:45–59	**232.2 (217.5–247.3)**	**139.5 (136.6–142.8)**	9.6 (7.1–12.1)	5.4 (3.0–7.9)	22.3 (19.8–24.9)	21.3 (15.5–26.4)	45.4 (41.2–49.3)	37.6 (32.4–42.7)	4.9 (2.7–6.7)	8.5 (6.4–10.3)	**36.7 (34.5–38.8)**	**31.6 (29.6–33.7)**	12.0 (10.4–14.2)	14.4 (11.5–17.6)
M:60 +	573.2 (558.3–588.2)	553.6 (542.6–567.4)	**72.7 (70.2–75.2)**	**34.8 (27.5–42.1)**	135.2 (132.7–138.0)	145.4 (136.3–157.1)	**181.1 (177.0–185.1)**	**165.4 (154.6–176.2)**	**51.4 (49.2–53.0)**	**65.5 (63.7–67.5)**	**128.7 (125.4–131.7)**	**114.9 (112.7–115.8)**	70.9 (69.0–73.2)	68.4 (65.3–72.1)
Tot M	**262.9 (245.3–276.5)**	**232.2 (230.5–235.2)**	**21.9 (15.6–26.2)**	**11.1 (9.9–12.2)**	44.5 (36.0–52.3)	45.4 (40.1–51.6)	72.4 (61.7–82.2)	63.1 (59.7–67.3)	15.5 (11.1–19.9)	14.8 (12.7–16.4)	60.4 (52.1–70.5)	56.1 (53.7–59.3)	23.5 (21.6–25.6)	29.8 (25.5–33.0)
**TOT**	**246.5 (232.0–259.5)**	**222.6 (219.7–226.5)**	**16.8 (14.5–18.7)**	**10.1 (8.0–12.0)**	26.5 (22.3–29.4)	37.6 (27.4–46.6)	61.8 (57.7–66.0)	65.2 (54.9–75.1)	**10.3 (9.2–11.2)**	**16.7 (14.4–18.5)**	58.1 (53.9–63.6)	55.1 (53.8–56.9)	**13.5 (11.5–15.8)**	**19.3 (16.8–22.3)**

Statistically significant differences are highlighted in bold

Considering subgroups by age, younger males and females with MS (20–44 years of age) had statistically significant higher rates of hypertension, stroke, and diabetes than the ones in the general population, whereas IHD was significantly higher only in males. The middle age MS group (45–59 years of age) had statistically significant higher rates of hypertension and COPD for both sexes and higher rates of stroke and IHD only in females. Among oldest group (> 60 years of age), PwMS had statistically significant higher rates of stroke for both sexes, and diabetes and COPD for males, whereas general population had statistically significant higher rates of heart failure for both sexes, and COPD and IHD for females only (Table 3).

## Discussion

Studies on comorbidities are of great and increasing interest because these conditions are common in PwMS and, if not identified, they can have a direct impact on the disease, affecting clinical disability, and an indirect one due to their role as confounding factor during different disease phases. First, during the diagnostic phase, concomitant diseases could influence symptoms at onset leading to misdiagnosis. Secondly, prior to start specific treatments, comorbid conditions should be considered in MS, as they can limit or negatively affect the utilization of some disease modifying drugs (DMDs), especially in case of polypharmacy. Third, during the follow-up period, they can lead to a worsening of the clinical status, with an impact on MS prognosis and patients’ autonomy, empowerment, and quality of life.

In this study, the prevalence rates of comorbidities resemble those of published data, with hypertension being the most frequent comorbidity (around 30%) followed by diabetes (6%) [10, 11]. Specifically, resulting data from the Tuscan cohort showed a greater risk of developing comorbidities in PwMS, especially in the young and intermediate groups, suggesting an important role of MS as risk factor in these age classes.

As expected, an increasing trend is observed in both study populations with advancing age [12–14] and comorbidities were more frequent in males both in PwMS and in the general population [10]. This sex difference reflects the same trend already present in the Italian population, which could partly explain the lower survival age of males (80.8 years) than females (85.2 years) [15].

The increased risk of chronic diseases in MS may be related to the higher frequency of adverse health behaviors in PwMS, such as higher prevalence of smokers and a lower level of physical activity in comparison with the general population [16] and a low adherence to healthy diet [17]. A possible further explanation for this finding is called the “surveillance hypothesis”, because patients undergo to tighter screening programmes than healthy individuals which can reveal conditions otherwise hidden. Indeed, PwMS receive frequent screening and surveillance tests before and during treatment to monitor the potential side effects of these therapies. As they are closely monitored, it is possible to find comorbidities earlier in these patients than in the general population of the same age [18].

Alternatively, the “competing demands hypothesis” should be considered [18], which draws attention to the misattribution of symptoms by clinicians of a new comorbidity to MS causing an underestimation of comorbidities’ diagnosis.

Looking at the correlations between MS and its comorbidities, patients had a higher risk of hypertension in the first two age groups in both sexes compared to general population, although an increasing trend with age was observed in both populations. Considering the total population, 25% of PwMS was affected by hypertension, in line with a previous American study showing the same prevalence [11]. Hypertension brings to an increased risk of complications and it could be linked, as other comorbidities, to autoimmunity and chronic inflammation of MS, in addition to other unhealthy lifestyles, such as a sedentary behavior, poor health, and socioeconomic status [19, 20].

Further, a higher prevalence of diabetes was observed in the younger age group of both sexes among PwMS than in the general population and, only in males, in the older age group. Unfortunately, with administrative data, it is not possible to differentiate the types of diabetes and consequently their distribution in different age and sex groups. Several studies showed that the autoimmune pathogenesis of MS might contribute to the predisposition to other comorbidities, including type 1 diabetes mellitus. Although type 1 diabetes accounts for only 10% of the total cases [21], this disease is of particular interest because it shares similar characteristics with MS, in terms of epidemiological distribution and aetiology. Indeed, they both showed a decreasing gradient of prevalence from north to south [22, 23], and they seem to have a common genetic risk [24]. In addition, another possible explanation is a similar viral origin [25, 26], in particular the association with the infection with Epstein-Barr virus [27, 28]. The correlation with MS and type 2 diabetes mellitus, on the other hand, could be explained not only with the unhealthy lifestyle in PwMS but also with the use of corticosteroid therapy [26, 29].

In our study, we found that the prevalence of stroke is higher in PwMS than in the general population irrespective of sex and age, in line with the literature [30, 31]. The risk of developing cerebrovascular disease has been evaluated in PwMS and, although there are discordant findings [32], it seems that there is an increased risk of stroke especially in the years immediately following the diagnosis of MS [33]. Causes of different nature have been suggested: surveillance hypothesis, misdiagnosis, utilization of high doses of corticosteroids for relapses [30], the higher presence of antiphospholipid antibodies in PwMS than in general population [33], and dysregulated inflammatory processes, characterizing MS, increasing risk of arterial atherosclerosis, and consequently of stroke [34].

When considering CV diseases, they must be taken into account as confounding factors themselves and as contraindication or side effect to the use of some DMDs [35]. In the present study, IHD prevalence was higher in females with MS in the middle age group and in males with MS in the younger age group, whereas is higher in females in the general population in the older age group. However, the prevalence was generally higher in males, both in MS and in the general population. Literature data regarding the association of IHD and MS are conflicting [35]: a recent review finds no increased risk in MS [36], whereas another study reveals an increased risk of IHD in males with MS under 60 years of age, confirming our results [35]. IHD in PwMS seems to be linked to an increased risk of complications of the clinical picture and a reduction in survival [35, 37]. Possible reasons of this association may be a poor physical activity in PwMS and/or the common presence of hypertension, hyperlipidemia, and diabetes as non-specific risk factors for cerebro-cardiovascular disease [35].

Regarding cardiac infarction only, data showed a higher prevalence in the total general population vs MS one. Literature data on myocardial infarction, on the other hand, are discordant, also in view of characteristics of the different populations under study. Moreover, several studies suggested that, in addition to the common risk factors, the inflammatory state (also common to other immune-mediated diseases) may independently increase the possibility of developing these diseases [38]. Concerning the risk of heart failure, this study did not show any differences between PwMS and general population in the younger and intermediate age groups for both sexes, while in the over 60 age group, there is an increased prevalence in the general population compared to MS one. These results are not confirmed in previous works: indeed, a study conducting on a large Danish MS cohort reveals a slightly higher risk of acute myocardial infarction, stroke, and heart failure within the first year after MS diagnosis in comparison with the general population. This increased risk persisted, during long-term follow-up, for stroke and heart failure [39].

Chronic pulmonary diseases, and COPD in particular, occur with high frequency as comorbidity in young PwMS [14]. In this study, we found an increased risk in people with MS in the young and intermediate age groups (statistically significant only in the second group). In the older age group, however, the same correlation was observed only in males, while in females, the general population was at greater risk than those with MS. The cause of this comorbidity probably lies both in the disability itself, which leads to a sedentary lifestyle. Disability is therefore the *primum movens* causing a series of consequences (principally an unhealthy lifestyle and a reduced physical exercise), which in PwMS could lead, in the long term, to a worsening of the clinical condition. In addition, the presence of chronic diseases of pulmonary origin may complicate or be a complication of some DMDs [14].

A strength of our study is the use of administrative data, which represent a cost- and time-saving tool for the identification of chronic disorders and their relationship with MS, essential for a targeted public health planning. This source of data is also a limitation of our study due to the nature of administrative data which are not collected for scientific purposes, and they do not include clinical information on patients which might allow further comparisons and consideration.

## Conclusions

In conclusion, our findings confirm that PwMS have an increased prevalence of comorbidities, which may impact their already complex clinical picture. The identification and management of other chronic diseases is particularly noteworthy, as it may improve the clinical management of PwMS in all disease stages.

Fortunately, many of these comorbidities are related to modifiable risk factors. Therefore, it should be of primary importance in clinical practice to routinely screen patients for chronic diseases and to educate PwMS to healthy lifestyles. A multidisciplinary team including the general practitioner is needed to assess and assist patients in order to improve their quality of life, reduce disability, and increase patients’ survival.

## Data Availability

Data presented here are available upon request to the corresponding author.
